# Charting the path forward in Southeast Asia: Learning from the COVID-19 vaccination challenges in six ASEAN countries

**DOI:** 10.7189/jogh.14.03016

**Published:** 2024-03-15

**Authors:** Jolene Yin Ling Fu, Muhammad Harith Pukhari, Kristine Alvarado Dela Cruz, Amin Soebandrio, Le Van Tan, Watsamon Jantarabenjakul, Anak Agung Sagung Sawitri, Napaporn Chantasrisawad, Sidney Yee, Ruifen Weng, Raghav Sundar, Chee Wah Tan, Lin-Fa Wang, I-Ching Sam, Barnaby Young, I Nyoman Sutarsa, Yoke Fun Chan

**Affiliations:** 1 Department of Medical Microbiology, Faculty of Medicine, Universiti Malaya, Kuala Lumpur, Malaysia; 2 Microbiology Department, Laboratory Research Division of the Research Institute for Tropical Medicine, Muntinlupa City, Philippines; 3 Department of Microbiology, Faculty of Medicine, University of Indonesia, Jakarta, Indonesia; 4 Oxford University Clinical Research Unit, Ho Chi Minh City, Vietnam; 5 Thai Red Cross Emerging Infectious Diseases Clinical Centre, King Chulalongkorn Memorial Hospital, Bangkok, Thailand; 6 Department of Public Health and Preventative Medicine, Faculty of Medicine, Udayana University, Bali, Indonesia; 7 Diagnostics Development (DxD) Hub, Agency for Science, Technology and Research (A*STAR), Singapore; 8 Department of Haematology-Oncology, National University Cancer Institute, Singapore; 9 Yong Loo Lin School of Medicine, National University of Singapore, Singapore; 10 Duke-NUS Medical School, Programme in Emerging Infectious Diseases, Singapore; 11 Singapore Infectious Diseases Clinical Research Network (SCRN), National Centre for Infectious Diseases, Singapore; 12 Department of Infectious Diseases, Tan Tock Seng Hospital, Singapore; 13 Lee Kong Chian School of Medicine, Nanyang Technological University Singapore, Singapore; 14 School of Medicine and Psychology, College of Health and Medicine, The Australian National University, Canberra, Australia

**Figure:**
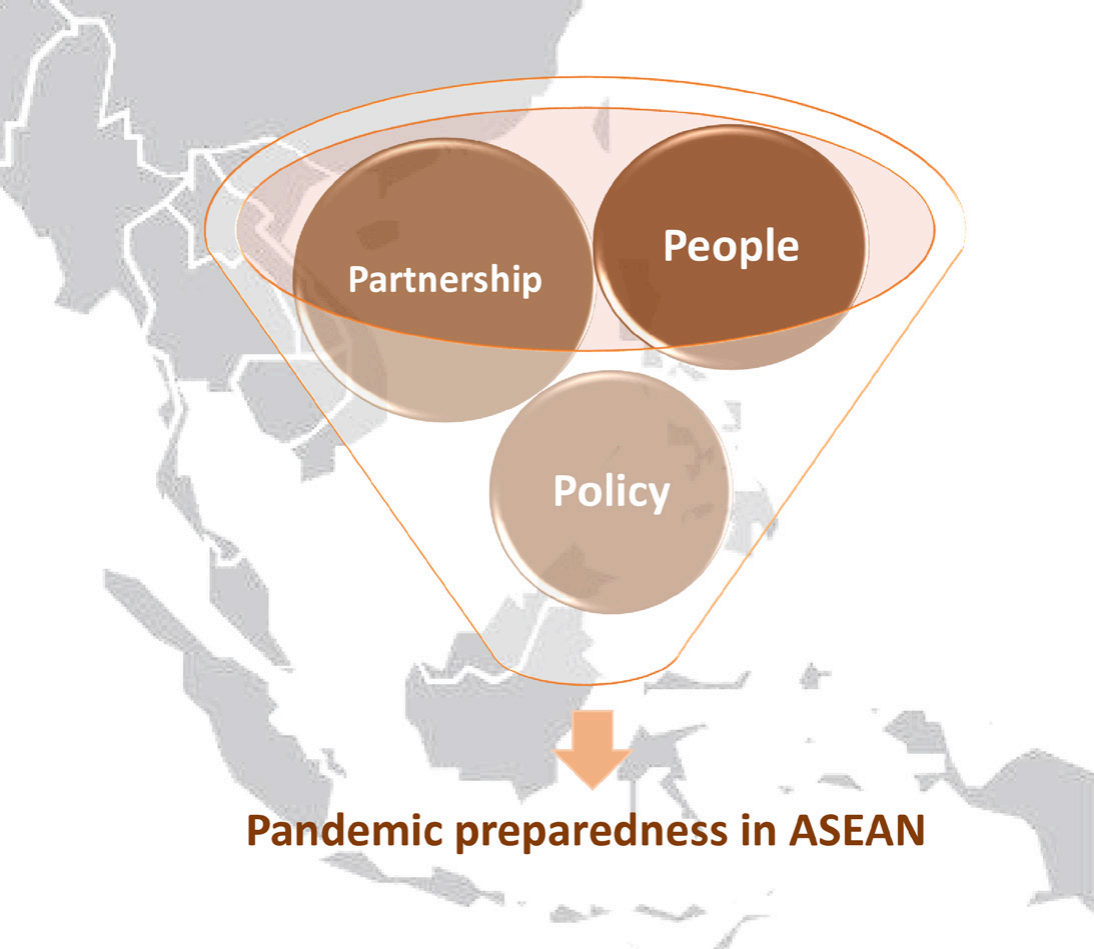
Photo: ASEAN preparedness for future pandemics with a focus on the 3P – People, Partnership and Policy. Underlying map of Asia: https://en.wikipedia.org/wiki/File:Blank_Asia.png#file, image in public domain (free to use, no specific license).

With a population exceeding 630 million (8% of the global population), the ten member states of the Association of Southeast Asian Nations (ASEAN) accounted for approximately 61 million (7.9%) of global COVID-19 cases and 808 166 (11.6%) of deaths, with case fatality rate (CFR) of 1.3% by the end of 2023 [1]. The actual figures are certain to be at least several-fold higher, with estimates suggesting an excess mortality of 1.2 million in the first two years of the pandemic. Despite the World Health Organization’s (WHO) 4 May 2023 declaration that coronavirus disease 2019 (COVID-19) is no longer a public health emergency, it remains a global threat. The global response to the COVID-19 pandemic has highlighted the importance of collaborative networks within ASEAN, particularly in scientific information, technology and research exchange, resource mobilisation, and capacity building. While individual ASEAN country implemented responses with varying degrees of success, a unified and cohesive regional approach is crucial for early variant detection, efficient resource allocation, and evidence-based public health policies. Without collaborative efforts, the collective regional response weakens, jeopardising the region's preparedness for future health crises.

In response to COVID-19, ASEAN launched several initiatives, including the ASEAN Comprehensive Recovery Framework (ACRF) [2], the ASEAN Travel Corridor Arrangement Framework, the ASEAN COVID-19 Response Fund, and the activation of the ASEAN Coordinate Council Working Group on Public Health Emergencies (ACCWG PHE) [3]. Although these initiatives contributed to mitigating the health, social, and economic impacts of the pandemic, the absence of a scientific network for COVID-19 serology surveillance in the ASEAN region hindered a thorough understanding of population susceptibility and the effectiveness of COVID-19 vaccination in the region. In May 2021, the ASEAN Committee of Science, Technology, and Innovation (COSTI) endorsed the establishment of the ASEAN Sero-Surveillance Study on COVID-19 vaccines (ASSeSS) Working Group, involving researchers from six ASEAN countries: Indonesia, Malaysia, Philippines, Singapore, Thailand, and Vietnam. The ASSeSS Working Group focused on evaluating vaccine efficacy, specifically monitoring antibody responses and immunoglobulin G avidity maturation over time. The local scientific data on the effectiveness of the various COVID-19 vaccines used in the region would serve as a guide in planning public health strategies in ASEAN. This article explores gaps in ASEAN’s scientific collaboration during the COVID-19 pandemic and highlight the importance of regional research networks such as ASSeSS for pandemic preparedness.

At the beginning of the COVID-19 pandemic, all member countries of the ASSeSS Working Group were spared from the explosive outbreaks observed in Western countries due to the timely implementation of strict public health and social measures, social distancing, border closure, and movement restrictions [4]. Experiences with previous outbreaks such as SARS-CoV in 2003 and bird flu (H5N1) in 2004, and a generally high level of trust in governments facilitated implementation and compliance with these preventative measures [5]. These strategies became less effective due to the emergence of highly transmissible variants of concern with relatively high rates of severe illness which threatened to overwhelm health care systems [6]. The strategies in ASEAN subsequently needed to pivot from containment measures to a large-scale vaccination programme.

**Figure 1:**
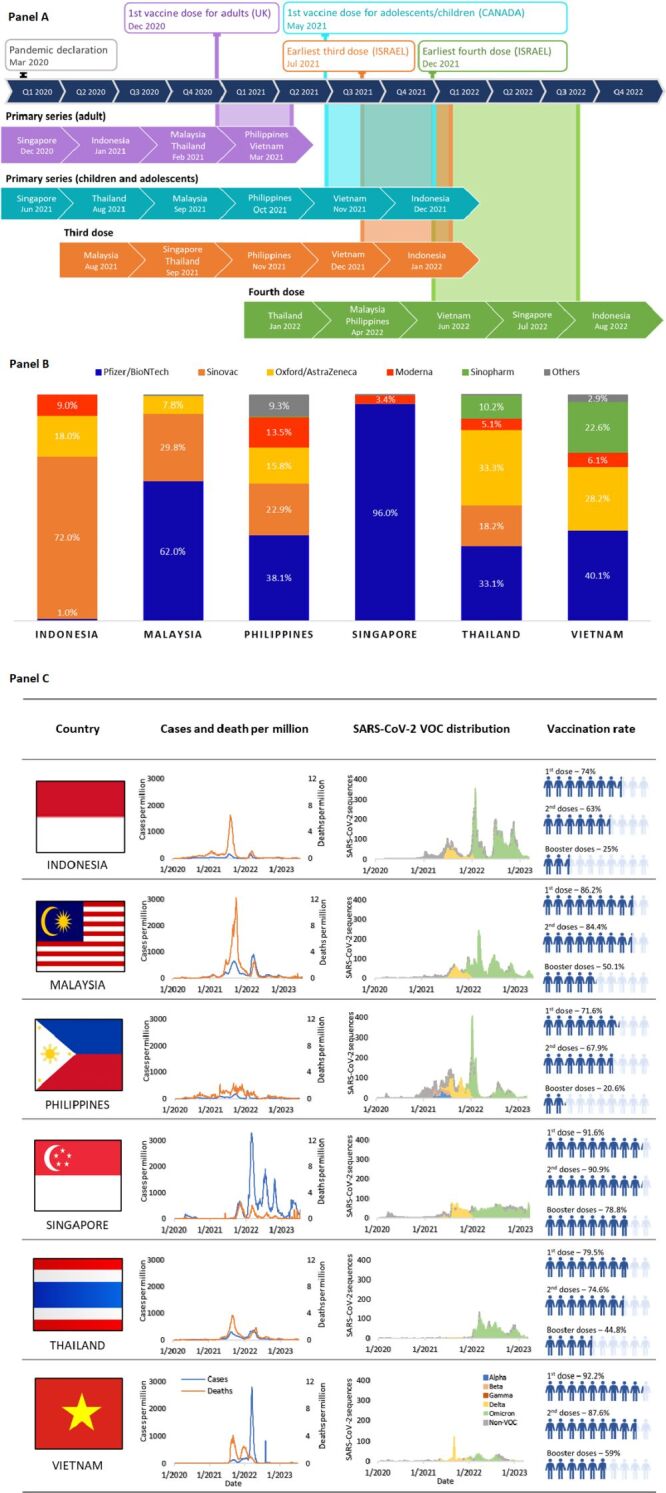
**Panel A.** Coronavirus disease 2019 (COVID-19) vaccination timelines and distribution including primary series in adults, children and adolescents, third doses and fourth doses, in six member countries of the ASSeSS Working Group (Indonesia, Malaysia, Philippines, Singapore, Thailand, and Vietnam). **Panel B.** Distribution of COVID-19 vaccines. We took the data from available public COVID-19 vaccine dashboards as of July 5, 2023. **Panel C.** Overview of COVID-19 situations including total cases and deaths per million, and SARS-CoV-2 variants of concern distribution from the beginning of the COVID-19 pandemic to 5 July 2023. Vaccination rates presented are as of 5 July 2023. We took the data from Our World in Data [8].

Due to limited or non-existent local vaccine production, ASEAN heavily relies on vaccine imports from the global marketplace, restricting choices of vaccines. In contrast, Western countries like the European Union pooled resources, shared risks, and coordinated efforts for a collective and equitable vaccination response, displaying a more unified approach than the initially independent ASEAN efforts. ASEAN strategies included direct purchase from suppliers (e.g. Singapore and Malaysia with Comirnaty Pfizer/BioNtech), bilateral donation (e.g. Vietnam and Indonesia; China donating and supplying Indonesia with CoronaVac (Sinovac) since December 2020), regional initiatives (e.g. Quad Vaccine Partnership mainly for Indonesia), and multilateral platforms (e.g. the COVID-19 Vaccines Advance Market Commitment (COVAX AMC) initiative donating vaccines to Cambodia, Indonesia, Laos, Myanmar, the Philippines, and Vietnam) [7]. Only recently some countries have begun increasing their local production capacity, for example, Indonesia with its ‘Merah Putih’ vaccine development. Despite the limitations, ASSeSS member countries have managed to procure vaccines at a competitive timeline compared to countries from other regions (**Figure 1**, panel A).

Logistical and infrastructural challenges related to vaccine distribution, storage, and transportation posed a great challenge to ASEAN at the beginning of the vaccination campaign. In the first six months of 2021, less than 5% of the ASEAN population was vaccinated. In addition, geographical, political and socioeconomic disparities contributed to an inequitable distribution of the vaccine in the region. These issues were especially prominent in ASEAN countries with lower Logistics Performance Index (LPI) scores, a benchmarking tool that measures the efficiency and effectiveness of logistic systems in different countries [9].

Regional cooperation and partnerships with international organisations, such as the WHO and Global Alliance for Vaccines and Immunization (GAVI) subsequently helped to improve vaccine access and distribution across ASEAN. Further, all member countries adopted a unique, vaccine-plus strategy, utilising a combination of COVID-19 vaccines to increase vaccination coverage (**Figure 1**, panel B). Mass vaccination efforts were carried out to increase vaccination coverage, either via large-scale vaccination centres, small-scale community-based vaccination drives, or mobile vaccination centres to reach rural regions.

To address challenges with vaccine hesitancy, ASEAN member countries adopted collaborative approaches that sought to engage all sectors of society. This included providing resources and funding needed for implementation of their strategies, and clear guidelines and recommendations for vaccine distribution. National task forces that have been established in each country to oversee pandemic responses helped to coordinate efforts across different sectors, including engagement of public health, medicine, economics, social work, private sector, and religious leaders. Decentralisation of decision-making to municipal governments promoted tailoring of strategies to address local challenges and limitations on handling COVID-19.

To date, COVID-19 cases and deaths are under control, and all member countries have successfully vaccinated 60–90% of their populations, while booster vaccination rates vary between 20.6 and 78.8% (**Figure 1**, Panel C). As the pandemic emergency recedes, ASEAN must conduct a risk assessment, and initiate preparations and strategic planning to predict, identify, and prevent outbreaks from escalating into pandemics, focusing on the 3P’s – partnership, people and policy. The ASSeSS Working Group has identified a few key areas of partnership to mitigate the effects of COVID-19 pandemic crises and prepare for future pandemics:

– enhance disease surveillance for early outbreak detection;

– establish and harmonise research tools such as virus, serum or cell line banks that will enable the rapid assessment of transmission dynamics and population susceptibility to emerging pathogens in the future;

– accelerate local medical diagnostics and equipment development with pilot production, quality assurance, regulatory clearance and scale-ups;

– adopt One Health and Planetary Health approaches to address human threats related to animals, climate, and the environment;

– establish formal and informal scientific networks for information and knowledge sharing to enable rapid response to outbreaks before they escalate into pandemics;

– collaborative funding to support efforts in pandemic preparedness, for example, a revolving fund for vaccine development and procurement for the ASEAN region could be established by fostering public and private collaborations [10].

With a large multi-ethnic population and rapidly developing economies, ASEAN is rich in diverse talent and expertise. Leaders plays a crucial role in combating future pandemics by establishing clear communication structures, promoting accountability, empowering people, and leveraging enabling technologies. Implementing policies to enhance communication, build public trust, and engage communities in pandemic management can further enhance our preparedness for future outbreaks.

Overall, ASEAN has demonstrated commendable efforts in managing the COVID-19 pandemic. We hold an optimistic outlook that ASEAN initiatives such as ASSeSS will champion pandemic preparedness by building basic research to translational research during peacetime and grow further in the future encompassing all the ASEAN member countries. Such an accomplishment would be remarkable, signifying a significant stride towards effectively tackling COVID-19 and future epidemics and pandemics. As epidemiologist, Larry Brilliant, who played a significant role in eradicating smallpox, aptly stated: ‘Outbreaks are inevitable, but pandemics are optional.’
